# Molecular classification of synovial sarcomas, leiomyosarcomas and malignant fibrous histiocytomas by gene expression profiling

**DOI:** 10.1038/sj.bjc.6600766

**Published:** 2003-02-18

**Authors:** Y-F Lee, M John, S Edwards, J Clark, P Flohr, K Maillard, M Edema, L Baker, D C Mangham, R Grimer, R Wooster, J M Thomas, C Fisher, I Judson, C S Cooper

**Affiliations:** 1 The Male Urological Cancer Research Centre, Institute of Cancer Research, 15 Cotswold Road, Belmont, Sutton, Surrey SM2 5NG, UK; 2 University of Michigan, Comprehensive Cancer Center, 6312 CCGC, 1500 East Medical Center Drive, Ann Arbor, MI 48109-0942, USA; 3 Department of Musculoskeletal Pathology, The Royal Orthopaedic Hospital NHS Trust, 111 Dale Road, Selly Oak, Birmingham B29 6AT, UK; 4 Sanger Centre, Wellcome Trust Genome Campus, Hinxton, Cambridge CB10 1SA, UK; 5 Department of Histopathology, The Royal Marsden NHS Trust, Fulham Road, London SW3 6JJ, UK

**Keywords:** synovial sarcoma, microarray, hierarchical clustering, leiomyosarcoma, malignant fibrous histiocytoma, soft tissue sarcoma

## Abstract

In this study, we have used genome-wide expression profiling to categorise synovial sarcomas, leiomyosarcomas and malignant fibrous histiocytomas (MFHs). Following hierarchical clustering analysis of the expression data, the best match between tumour clusters and conventional diagnosis was observed for synovial sarcomas. Eight of nine synovial sarcomas examined formed a cluster that was characterised by higher expression of a set of 48 genes. In contrast, sarcomas conventionally classified as leiomyosarcomas and MFHs did not match the clusters defined by hierarchical clustering analysis. One major cluster contained a mixture of both leiomyosarcomas and MFHs and was defined by the lower expression of a set of 202 genes. A cluster containing a subgroup of MFHs was also detected. These results may have implications for the classification of soft tissue sarcomas, and are consistent with the view that sarcomas conventionally defined as MFHs do not represent a separate diagnostic category.

Adult soft tissue sarcomas are malignant tumours that occur in supporting connective tissues throughout the body, other than the bone or cartilage. They account for around 1% of all cancers and 2% of cancer deaths with metastasis leading to death in about half of the cases (Dirix and van Oosterom, 1999; Singer *et al*, 2000; Weiss and Goldblum, 2001). These tumours are heterogeneous and a major complication in the management of this disease is that a definitive classification scheme has been slow to emerge. Synovial sarcomas are relatively well defined with two clear subcategories designated monophasic and biphasic, distinguishable following immunohistochemical examination (Fisher, 1998). The majority of cases of synovial sarcoma contain a t(X;18)(p11.2;q11.2) translocation that results in the fusion of the chromosome 18 gene *SYT* to three closely related genes *SSX1, SSX2* and *SSX4* on the X chromosome (Clark *et al*, 1994; Crew *et al*, 1995; Skytting *et al*, 1999). However, this tumour is of uncertain histogenesis and despite its name, it does not appear to originate from the synovium. Leiomyosarcomas are malignant tumours with smooth muscle differentiation that are defined as a single group based on morphology and immunohistochemical examination. However, they exhibit a wide range of clinical behaviour that appears to be partly related to their site of occurrence, with tumours of the retroperitonium having poorer prognosis than tumours of the uterus and extremities (Weiss and Goldblum, 2001). Malignant fibrous histiocytoma (MFH) until recently was a major diagnostic category for sarcomas. However, the perceived absence of defining clinical or histopathological criteria has led to the proposal that MFH is not a single entity but rather a heterogeneous collection of poorly differentiated sarcoma types, the majority of which might be suitably recategorised into other tumour groups, including leiomyosarcomas, if suitable markers were in hand (Fletcher, 1992).

Since the overall behaviour of a cancer must be determined by the expression of the genes within it, it should be possible to use cDNA microarray technology to classify tumours and identify sets of genes whose expression define individual tumour groups. This approach has, for example, already been used by Alizadeh *et al* (2000) to identify two new subgroups of diffuse large B-cell lymphoma that had distinct clinical behaviour, and by Golub *et al* (1999) to distinguish acute myeloid leukaemia (AML) and acute lymphoblastic leukaemia (ALL). Recently, Nielsen *et al* (2002) have used data obtained using microarrays to molecularly characterise soft tissue tumours. These results showed that synovial sarcomas, gastrointestinal stromal tumours (GISTs), neural tumours and a subset of leiomyosarcomas showed distinct gene expression patterns. In the current study, we have used cDNA microarrays to investigate the gene expression profiles for synovial sarcomas, MFHs and leiomyosarcomas. Our result also show that synovial sarcomas clustered together exhibiting a characteristic gene expression profile, but the set of genes whose increased expression define this group is quite distinct from that described by Nielsen *et al* (2002). In addition, we have identified a second major cluster of tumours that contains both MFHs and leiomyosarcoma and we provide evidence for the existence of a subset within the MFH tumour category.

## MATERIALS AND METHODS

### Tumour and control RNA preparation

Sarcoma tissues were collected from patients undergoing surgery. Diagnoses were carried out by pathologists using conventional criteria, immunohistochemistry and electron microscopy. The tumour samples were snap-frozen in liquid nitrogen and stored at −80°C until RNA extraction. As a common reference sample in hybridisations, total RNAs from a combination of six cell lines including three sarcoma cell lines (HTB-175, HTB-115, CCL-121, CCL-224, T91-95 and HB4a) were used. HTB-175 (small cell lung cancer), HTB-115 (leiomyosarcoma), CCL-121 (fibrosarcoma) and CCL-224 (colorectal adenocarcinoma) were obtained from the American Type Culture Collection. T91-95 (alveolar rhabdomyosarcoma) was obtained from Dr T Gordon (Institute of Cancer Research, Sutton, UK). HB4a (immortalised human mammary luminal epithelial cell line) was obtained from Dr MJ O'Hare. Cells were grown according to the suppliers' instruction. Total RNA was extracted from the tumours and cell lines by the TRIZOL® method (GibcoBRL, Invitrogen, Paisley, UK).

### cDNA microarray slide preparation and RNA labelling

Microarray slides were gridded with the ‘ICR-geneset’ that consisted of 5603 I.M.A.G.E cDNA clones (including 169 duplicates) acquired from the UK Human Genome Mapping Project Resource Centre and Research Genetics (http://www.resgen.com). Information on the geneset can be found at http://www.icr.ac.uk/array/array.html. The preparation of the microarray slides including gridding and blocking were as described in Clark *et al* (2002).

Total RNA was labelled by reverse transcription using Superscript II (Invitrogen, Paisley, UK) using Cy5- or Cy3-labelled dCTP. Cy5 was used for labelling tumour RNA samples, while Cy3 was used for labelling control cell line pool RNA. Total cellular RNA (4 *μ*g) was reverse transcribed overnight at 37°C with 400 U Superscript II (Invitrogen) with 500 *μ*M dGTP, dATP and dTTP, 200 *μ*M dCTP, 100 *μ*M DTT, 100 *μ*M Cy5- or Cy3-labelled dCTP (Amersham, Bucks, UK) and 30 *μ*M random primer (5′-IIINNNNNN-3′, where I is inosine) (100×excess) in a 20 *μ*l reaction of 1×first-strand buffer (Invitrogen). Labelling reaction was stopped by the addition of EDTA (pH 8.0) to 90 mM. Cot-1 DNA (12.5 *μ*g) (Invitrogen) was added. The sample was heated at 70°C for 10 min in 40 mM NaOH. 0.5×SSPE (Sigma, Dorset, UK) (400 *μ*l) was added and the sample was filtered through a 0.1 *μ*M ultrafree-MC filter column (Millipore, Billerica, MA, USA). Sample volume was then reduced to 30 *μ*l using a Microcon YM-30 filtration unit (Millipore). A 400 *μ*l volume of 0.5× SSPE (Sigma, Dorset, UK) was added and this process was repeated twice with a final reduction in volume to 15 *μ*l.

### Microarray hybridisation

Prehybridisation of the microarray slide was performed by adding 500 *μ*l of prehybridisation mix (6×SSPE pH 7.4, 12.5 mM EDTA pH 8.0, 0.1% (vv^−1^) Tween 20) on the slide and incubating at 65°C overnight in a sealed humid box. The microarray slide was then washed in 4×SSPE, 10 mM EDTA for 1 min; 2×SSPE, 10 mM EDTA for 1 min; and 0.1×SSPE for 1 min and drained on a rack. The microarray slide was then submerged in 70% (vv^−1^) deionised formamide, 2×SSC pH 7.0 at 65°C for 1 min (denaturation). Slides were then rinsed twice with 70% ethanol, then with 80 and 100% (vv^−1^) ethanol, blown dry with canned air (RS Components, Northants, UK) and prewarmed to 37°C in a hybridisation chamber (BDH Precision Engineers, Cambridge, UK) for 30–60 min. The labelled samples (from 4 *μ*g each of total RNA from tumour and control pool) were made up to 50 *μ*l in hybridisation mix (6×SSPE pH 7.4, 12.5 mM EDTA pH 8, 0.1% (vv^−1^) Tween 20). This mixture was heated to 99°C for 2 min and then at 65°C for 3 h. The mixture was then filtered through a 0.1 *μ*M ultrafree-MC filter column (Millipore, Billerica, MA, USA). The filtrate was heated to 99°C for 2 min, then at 65°C for 10 min, and 37°C for 10 min, pipetted onto a microarray slide and covered with a Hybrislip (22 mm×60 mm, Sigma, Dorset, UK). 6× SSPE (300 *μ*l) was pipetted underneath the slide, then the hybridisation chamber was sealed and incubated at 65°C overnight. The slide was then soaked in 4×SSPE, 10 mM EDTA for 1 min at 32°C until the coverslip fell off; and then washed with 4×SSPE, 10 mM EDTA for 1 min at 32°C; 2×SSPE, 10 mM EDTA for 1 min at room temperature and 0.1×SSPE for 1 min at room temperature. The slide was then dried with canned air.

Hybridised microarray slides were scanned in a GenePix 4000A scanner (Axon Instruments, Foster City, CA, USA). Slides were scanned at photomultiplier tube (PMT) voltage levels that provided a Cy5 : Cy3 hybridisation ratio across the slide of roughly 1. Ratios of fluorescent intensities (Cy5 : Cy3) for individual cDNA were then determined after subtraction of background using the GenePix Pro 3.0 software (Axon Instruments, Foster City, CA, USA).

### Analysis of microarray data

The scanned image was analysed with the GenePix Pro 3.0 software (Axon Instruments, Foster City, CA, USA). Fluorescent signals for both channels of the spots were determined. A local background in each channel was also determined for each spot, which is the median fluorescence of pixels in a halo surrounding the same array spot. Spots or areas of array with defects were flagged bad and were excluded from subsequent analysis. To enhance the reliability of the expression data, another round of quality filtering was performed. Spots with fluorescent spot intensity in each channel that were more than 1.4 times the local background (medians) of that channel were considered well measured (Alizadeh *et al*, 2000), and the data were further filtered to include only these spots. The median background intensity was subtracted from the median spot intensity to generate the background-corrected signal intensity for use in further analysis.

Further analyses including cluster analysis were performed using the GeneSpring software (Silicon Genetics, Redwood City, CA, USA). Fluorescent intensity ratios of Cy5 : Cy3 for individual spots of the filtered data were determined by dividing the background-corrected intensity for the Cy5 by that of the Cy3 channel. These ratios were then normalised by making the median of all measurements in each sample to be 1. The resulting ratios were further normalised so that the median of all measurements taken for a particular gene is 1. In order to better explore the differences between the samples, a subset of genes showing normalised expression ratios of above 2 in at least three of the samples or below 0.5 in at least three of the samples were selected. Hierarchical clustering was then applied to the log-transformed data for these genes, using average-linkage clustering with Pearson's correlation as the similarity metric. To ensure that potentially important genes were not excluded, the selection criteria used was slightly less stringent than those adopted by Nielsen *et al* (2002): their clustering studies were carried out on a subset of genes with an absolute value of fluorescence ratio at least three times greater than the geometric mean ratio of specimens looked at, in at least two arrays.

## RESULTS

### Hierarchical clustering of soft tissue sarcoma expression profiles

**Figure 1:**
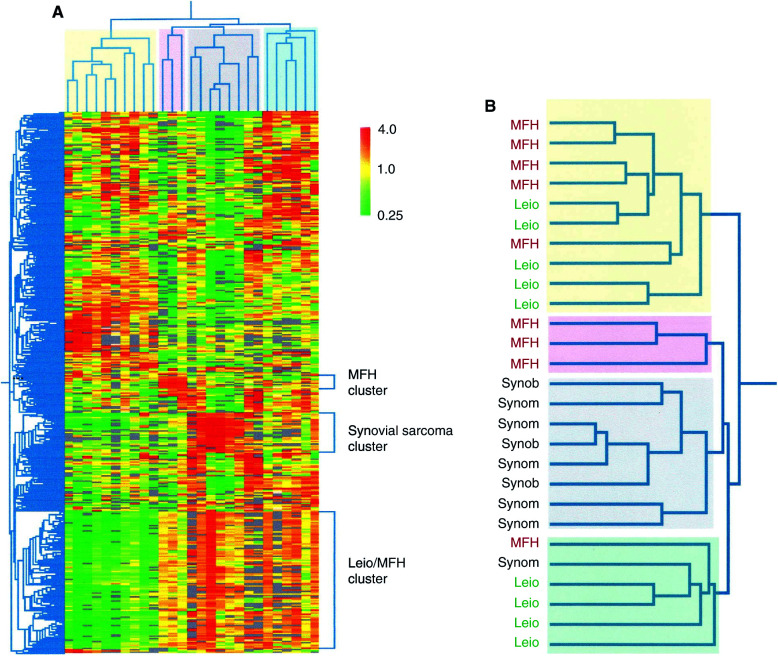
(**A**) Two-dimensional cluster analysis of soft tissue sarcomas (horizontal) and 833 genes (vertical). Each column corresponds to a tumour, and each row corresponds to a gene. Red indicates overexpression relative to the median expression of all the samples, while green indicates underexpression. Grey indicates missing or excluded data. The dendrogram at the top (tumour clustering) shows the degree of similarity of expression pattern between the tumours, and the dendrogram on the side (gene clustering) shows the degree of similarity of expression pattern between the genes across the tumour samples. The shorter the branches, the more similar are the two joined tumours or genes (**B**) Magnified tumour dendrogram showing the type of tumour. Abbreviations: MFH, malignant fibrous histiocytoma; Leio, leiomyosarcoma; Synom, monophasic synovial sarcoma; Synob, biphasic synovial sarcoma.

cDNA microarrays containing 5603 I.M.A.G.E. cDNA clones (Clark *et al*, 2002) were used to obtain expression profiles for 27 soft tissue sarcomas including nine synovial sarcomas, nine leiomyosarcomas and nine MFH tumours. We had previously established the high reliability of these microarray procedures for identifying overexpressed genes (Clark *et al*, 2002). In each experiment, Cy5-labelled sarcoma cDNA was cohybridised with Cy3-labelled reference cDNA from pooled human cell lines that served as an internal standard for the comparison of different experiments. Following filtering and normalisation, average-linkage hierarchical clustering analysis was performed on the data set. As we were specifically interested in the expression differences that may exist between the different sarcomas, a subset of 833 genes that showed the most variation in expression among the tumours was used in the cluster analysis. The resulting dendrogram is shown in Figure 1

. The tumours separated into four clusters. Interestingly, the clustering of the synovial sarcomas corresponded best to histological diagnosis. Eight of nine synovial sarcomas clustered together in a distinct group. A second cluster was composed of a mixture of five leiomyosarcomas and five MFH tumours. The third cluster contained a small subset of MFHs, while the fourth group contained mostly leiomyosarcomas (four) together with an MFH and the outlying synovial sarcoma.

### Gene clustering analysis

Gene clustering groups together genes whose expression patterns vary in a similar way among the sarcomas examined (Figure 1, vertical axis). In particular, these analyses identified a synovial sarcoma cluster of 48 sequences representing 44 different genes that appeared to have increased expression in synovial sarcoma compared to leiomyosarcomas and MFHs (Table 1

**Table 1:** Synovial sarcoma gene cluster

**Clone ID**	**UG cluster^a^**	**Symbol**	**Gene product**
245330	Hs.349109	*IGF2*	Insulin-like growth factor 2
245330	Hs.349109	*IGF2*	Insulin-like growth factor 2
754406	Hs.172631	*ITGAM*^b^	Integrin, *α* M
754406	Hs.172631	*ITGAM*^b^	Integrin, *α* M
2242404	Hs.105445	*GFRA1*^b^	GDNF family receptor *α* 1
383188	Hs.80539	*RCV1*	Recoverin
48799	Hs.78854	*ATP1B2*^b^	ATPase, Na^+^/K^+^ transporting, *β* 2 polypeptide
197520	Hs.225977	*NCOA3*^b^	Nuclear receptor coactivator 3
742082	Hs.1872	*PCK1*^b^	Phosphoenolpyruvate carboxykinase 1
950680	Hs.108327	*DDB1*	Damage-specific DNA binding protein 1
594633	Hs.5338	*CA12*^b^	Carbonic anhydrase XII
145112	Hs.168383	*ICAM1*^b^	Intercellular adhesion molecule 1 (CD54)
725473	Hs.16349	*KIAA0431*^b^	KIAA0431
85259		b	Genomic matching AL137072 at 9q31.1
232933	Hs.10755	*DPYS*^b^	Dihydropyrimidinase
297061	Hs.10755	*DPYS*^b^	Dihydropyrimidinase
753923	Hs.1334	*MYB*^b^	MYB transcription factor
80910	Hs.183556	*SLC1A5*^b^	Solute carrier family member 5
427750	Hs.1985	*SPTA1*^b^	Spectrin, *α*, erythrocytic 1
384078	Hs.106876	*ATP6V0D1*^b^	ATPase, H+ transporting V0 subunit d isoform 1
758343	Hs.173125	*PPIF*^b^	Peptidylprolyl isomerase F
46196	Hs.19261	*DYT1*^b^	Dystonia 1
884283	Hs.283742	b	Retrotransposon
42331	Hs.91916	*clone 23892*^b^	Hypothetical protein
267865		b	Genomic matching AC104070 at 4q35.2
795325			Genomic matching AL049796 at 1p22-31.1
211548	Hs.73858	*CPN2*	Carboxypeptidase N, polypeptide 2
1589786	Hs.111	*FGF9*^b^	Fibroblast growth factor 9
210575	Hs.2288	*VSNL1*^b^	Visinin-like 1
277507	Hs.75652	*GSTM5*^b^	Glutathione *S*-transferase M5
52430	Hs.23106	*TRAP100*^b^	Thyroid hormone receptor-associated protein (100 kDa)
756968	Hs.144700	*EFNB1*^b^	Ephrin-B1
502333	Hs.49765	*VLCS-H1*^b^	Very long chain acyl-CoA synthetase homolog1
135085	Hs.154782	*AP3S2*	Adaptor-related protein complex 3, *σ* 2 subunit
270626	Hs.106415	*PPARD*^b^	Peroxisome proliferative activated receptor, *δ*
251019	Hs.194657	*CDH1*^b^	Cadherin 1 , E-cadherin
291290	Hs.278632	*SSX4*^b^	Synovial sarcoma, X breakpoint 4
727251	Hs.1244	*CD9*	CD9 antigen
49959	Hs.179747	*EVI5*	Ectopic viral integration site 5
809598	Hs.151051	*MAPK10*	Mitogen-activated protein kinase 10
23173	Hs.151051	*MAPK10*	Mitogen-activated protein kinase 10
221846	Hs.211773	*CHES1*^b^	Checkpoint suppressor 1
177737	Hs.29117	*PURA*^b^	Purine-rich element-binding protein A
773554	Hs.289043	*SPIN*^b^	Spindlin
813742	Hs.82848	*SELL*	Selectin L
797048	Hs.68879	*BMP4*^b^	Bone morphogenetic protein 4
813520	Hs.372513	*GYPB*^b^	Glycophorin B
233078	Hs.11711	*KIAA0329*^b^	KIAA0329

a DNA sequencing was used to confirm the identity of all genes in this table.

b Genes with levels of expression statistically significant different (corrected *P*=0.01) in synovial sarcomas compared to sarcomas in other groups by Wilcoxon – Mann – Whitney test utilising Benjamini and Hochberg false discovery rate to correct for multiple testing.

). Each of the 48 sequences in this cluster was resequenced to confirm its identity. Application of the Wilcoxon – Mann – Whitney test utilising the Benjamini and Hochberg false discovery rate to correct for multiple testing (corrected *P*=0.01) confirmed that 36 of these showed statistically significantly different levels of expression in synovial sarcomas compared to sarcomas in other groups. The single synovial sarcoma that had a very different expression profile from other cases of synovial sarcoma was excluded from this analysis.

The observation that *SSX4* is included within the synovial sarcoma gene set flagged in these analyses demonstrates proof of principle. Fusion of *SYT* to *SSX1*, *SSX2* and *SSX4* causes inappropriate transcription of *SSX* sequences in synovial sarcoma that is characteristic of this tumour group (Crew *et al*, 1995; Skytting *et al*, 1999). Although *SSX1* and *SSX2* were not present on the microarray, they contain significant regions that exactly match *SSX4* sequences and their transcripts would be likely to crossreact with *SSX4* in these microarray studies. The genes present in the synovial sarcoma represent many functional groups. For example, they included genes implicated in embryonic development (*FGF9*), transcriptional regulation (*SSX4*, *NCOA3*), cell signalling (*EFNB1*) and cellular adhesion (*CDH1*, *ICAM1*). Interestingly, a gene encoding receptor for the drug cyclosporin A (*PPIF*) was overexpressed in synovial sarcoma.

The MFH subgroup appeared to be characterised by the increased expression of a set of 21 genes (Table 2

**Table 2:** Malignant fibrous histiocytoma gene cluster

**Clone ID**	**UG cluster^a^**	**Symbol**	**Gene product**
75254	Hs.10526	CSRP2	Cysteine- and glycine-rich protein
74566	Hs.79090	*XPO1*	Exportin 1
898198	Hs.43910	*CD164*	CD164 antigen
897806	Hs.197540	*HIF1A*	Hypoxia-inducible factor 1, *α* subunit
789376	Hs.13046	*TXNRD1*	Thioredoxin reductase 1
46182	Hs.251871	*CTPS*	CTP synthase
897906	Hs.74101	*SYK*	Spleen tyrosine kinase
773192	Hs.78580	*DDX1*	DEAD/H box polypeptide 1
730410	Hs.1765	*LCK*	Lymphocyte-specific protein tyrosine kinase
741497	Hs.204238	*LCN2*	Lipocalin 2
212165	Hs.146354	*PRDX2*	Peroxyredoxin 2
1031552	Hs.3796	*EPHB6*	Eph kinase
782811	Hs.139800	*HMGIY*	High mobility group isoforms I and Y
840364	Hs.172673	*AHCY*	*S*-adenosyl homocysteine hydrolase
814989	Hs.17883	*PPMIG*	Protein phosphatase 1G
487373	Hs.80986	*ATP5G1*	ATP synthase
36393	Hs.278544	*ACAT2*	Acetyl-coenzyme A acetyl transferase 2
325062	Hs.78452	*SLC20A1*	Solute carrier family 20 member 1
1493390	Hs.75117	*ILF2*	Interleukin enhancer-binding factor 2
781341	Hs.1197	*HSPE1*	Heat shock 10 kDa protein 1 (chaperonin 10)
1325605	Hs.169248	*HCS*	Cytochrome *c*

a DNA sequencing was used to confirm the identity of all genes in this table.

). This subgroup is potentially very interesting despite its small size (three tumours), particularly when considered together with the existence of the mixed leiomyosarcoma/MFH cluster. Further analysis of a larger set of MFHs may still be required to confirm its existence and to assess the significance of these genes. The hierarchical cluster that contained five leiomyosarcomas and five MFHs was characterised by low expression of a set of 202 genes (Figure 1). These genes are listed at http://www.icr.ac.uk/array/array.html (Table A).

## DISCUSSION

Genome-wide analysis of gene expression using microarray technology is proving an important aid in the molecular diagnosis and classification of human malignancies including leukaemias and lymphomas (Golub *et al*, 1999; Alizadeh *et al*, 2000), breast cancer (Perou *et al*, 2000; Sorlie *et al*, 2001) and melanoma (Bittner *et al*, 2000). In the current study, we have used a cDNA microarray technique to obtain expression profiles for three diagnostic categories of adult soft tissue sarcomas: synovial sarcomas, leiomyosarcomas and MFHs. Our results show that most (eight out of nine) synovial sarcomas clustered as a single group based on their gene expression portrait and that this cluster was characterised by the raised expression of a set of 48 genes. Nielsen *et al* (2002) obtained expression profiles of 41 sarcomas including eight synovial sarcomas using either 22 or 42k gene microarrays. In common with our analyses this group found that synovial sarcomas exhibited a distinct expression profile and identified a set of genes (104 genes, representing 89 different genes) whose overexpression appeared to be characteristic of synovial sarcomas. Remarkably, there was very little overlap between the genes in this set and those in our synovial sarcoma gene cluster: indeed the only gene in common was *SSX4*. It is noteworthy that only 20 of the 44 distinct genes listed in Table 1 were included in the genes used by Nielsen *et al* (2002) for their clustering analyses of 41 sarcomas and that only 11 of the 89 distinct genes in the synovial sarcoma cluster of Nielsen *et al* (2002) were present in the set of 833 genes selected for our clustering studies. The lack of correlation may also partly reflect the need to examine much larger series of individual tumours in microarray studies before coming to a firm conclusion on the identity of the genes whose over or underexpression define a tumour group. This is illustrated by the gene *ATP1B2* (Table 1) that had consistently increased expression in our eight clustered synovial sarcomas, but was probably not selected by Nielsen *et al* (2002) because elevated expression was observed in a lower proportion of their synovial sarcomas. Conversely *BMP7*, present in the synovial sarcoma cluster of Nielsen *et al* (2002), was upregulated in only a low proportion of our synovial sarcomas. Other genes, such as *GFRA1*, *BMP4* and *IGF2* that were present in our cluster, were probably not selected by Nielsen *et al* (2002) because they were also upregulated in GIST tumours, a category not examined in our study. *EGFR* present in the synovial sarcoma cluster of Nielsen *et al* (2002) was not selected in our analysis because, although upregulated in five of six clustered synovial sarcoma that had data for this gene, it was also upregulated in one leiomyosarcoma and four MFHs. It is also noteworthy that in contrast to the study of Nielsen *et al* (2002), our analysis did not distinguish two separate groups of leiomyosarcomas. This might be related to the fact that only seven of the genes (24 clones representing 20 different genes) that distinguished their ‘calponin’ subgroups were present in the group of 833 genes used in our clustering studies.

We have identified a mixed cluster containing both MFH tumours and leiomyosarcomas that was characterised by low expression of a set of 202 sequences. When considered together with our preliminary evidence for a subcategory of MFH tumours, this observation could be considered to support the proposal of Fletcher (1992) that MFH does not exist as a single diagnostic category and that many MFH should be reclassified into groups with other soft tissue sarcomas, including leiomyosarcomas.

During immunohistochemical diagnosis the detection of markers such as keratin, epithelial membrane antigen (EMA) and Bcl2 are characteristic of synovial sarcoma (Fisher, 1998). In the current study, we have confirmed that analysis of microarray expression profiles can also be used to group most synovial sarcomas in a single cluster. In addition, groups of genes that could potentially be used in differential diagnosis or have implications in the development of sarcoma were identified. We have also provided evidence on heterogeneity of MFH from a gene expression perspective that may aid in the development of a definitive classification scheme for soft tissue sarcomas.
